# Streptococcus oralis-Induced Meningoencephalitis and Ventriculitis in a Geriatric Female Patient

**DOI:** 10.7759/cureus.46101

**Published:** 2023-09-27

**Authors:** Mohammed Adly, Amr Elmoheen, Mohamed M Helmi Ahmed, Abdelraheem Hanbouly, Larissa Michael Mishreky

**Affiliations:** 1 Emergency Department, Hamad Medical Corporation, Doha, QAT; 2 Medicine, Qatar University, College of Medicine, Doha, QAT; 3 Citation Clinical Imaging Department, Women's Wellness and Research Center (WWRC), Hamad Medical Corporation, Doha, QAT

**Keywords:** emergency medicine, meningitis, meningoencephalitis, streptococcus oralis, streptococcus oralis induced meningoencephalitis

## Abstract

A 71-year-old female with a history of hypertension, diabetes, and dyslipidemia developed altered mental status, fever, headache, and vomiting. Subsequent evaluation revealed meningoencephalitis and ventriculitis due to Streptococcus oralis, which was found to be ceftriaxone-sensitive. The patient's condition improved with ceftriaxone treatment, leading to complete recovery. This case underscores the significance of including Streptococcus oralis in the differential diagnosis of meningitis or encephalitis.

## Introduction

Meningoencephalitis, an inflammation involving both the meninges and the brain tissue, can be triggered by various infectious agents, encompassing bacteria, viruses, fungi, and parasites [1]. Among these, Streptococcus oralis, a common resident of the oropharyngeal, gastrointestinal, and genitourinary microbiota, is typically characterized by its low pathogenic and virulent nature. Notably, it is often implicated in cases of subacute infective endocarditis [2]. Nevertheless, there have been sporadic instances where this bacterium has been pinpointed as the causative agent of meningoencephalitis. This serves as a crucial reminder of the potential of this organism to instigate severe neurologic infections despite its usual benign presence in the human body.

## Case presentation

A 71-year-old woman with hypertension, diabetes mellitus, and dyslipidemia was brought to the emergency department after experiencing altered mental status, fever, headache, and vomiting for a day.

On examination, she had a temperature of 39°C, heart rate of 125 bpm, respiratory rate of 20 bpm, and blood pressure of 170/80 mmHg. Her oxygen saturation on room air was 96%, and random blood glucose was 12 mmol/L. She appeared confused with a Glasgow Coma Scale score of 11. Her pupils were equal and reactive, cranial nerves II-XII were intact, and neck stiffness was noted. There was no focal weakness, sensory deficits, or signs of raised intracranial pressure. Motor functions were intact, and reflexes were symmetrical and normal. Her chest and heart examination was unremarkable, and no skin rash was detected.

Laboratory findings included a white blood cell count of 7.5 × 10^3/μL, hemoglobin of 12.4 g/dL, and platelet count of 158 × 10^3/μL. Venous blood gas analysis showed a pH of 7.38, partial pressure of carbon dioxide (PaCO2) of 45 mmHg, and bicarbonate (HCO3) of 26 mEq/L. Serum glucose was 12 mmol/L, lactate 3.4 mmol/L, and the International Normalized Ratio (INR) 1.1. Kidney and liver function tests were within normal limits.

Empirical treatment for meningitis was initiated with ceftriaxone 2 grams, vancomycin 1 gram, acyclovir 800 mg, and dexamethasone 8 mg intravenously. The head Computed Tomography (CT) scan was unremarkable. Lumbar puncture showed cloudy cerebrospinal fluid (CSF) with a white blood cell count of 20,000/μL, red blood cell count of 1111/μL, glucose concentration of 6.95 mmol/L, and protein concentration of 4.61 g/L. CSF culture remained negative, but blood culture grew Streptococcus oralis, which was susceptible to ceftriaxone.

**Figure 1:**
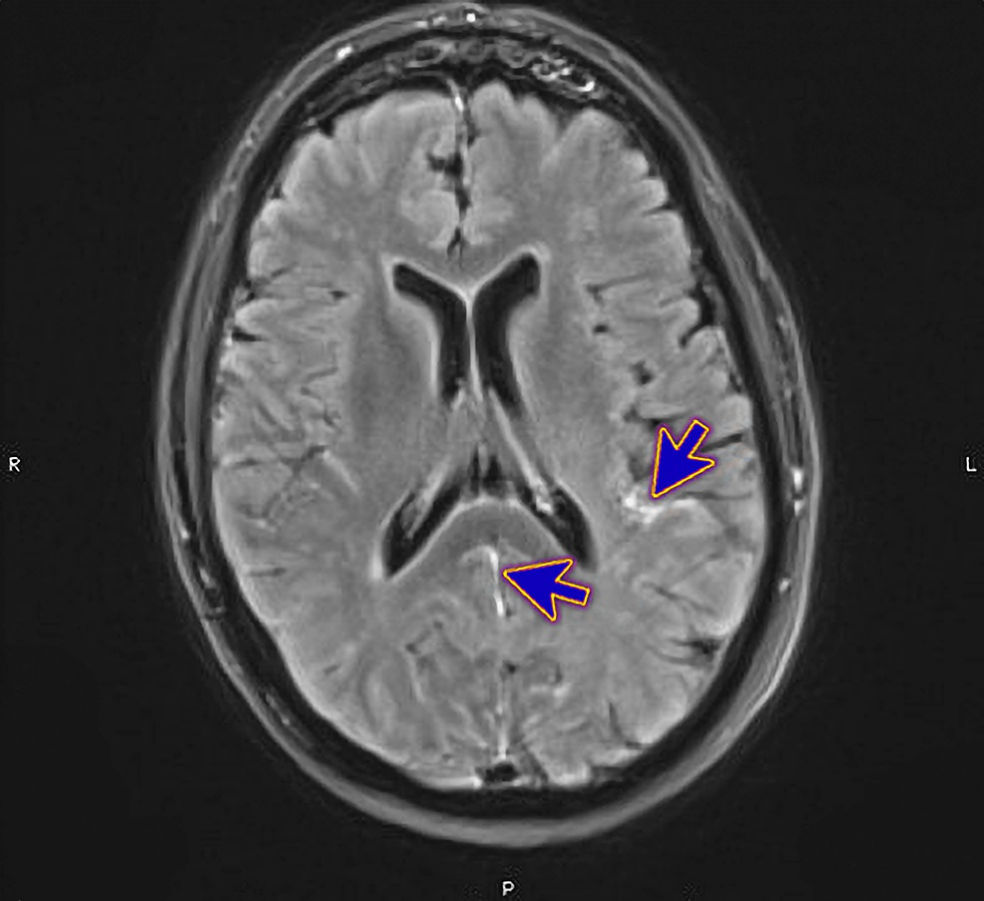
Magnetic Resonance Imaging (MRI) of the brain showing frontoparietal leptomeningeal enhancement along with interpedicular involvement with suspected cervical ependymal enhancement (blue arrows)

**Figure 2:**
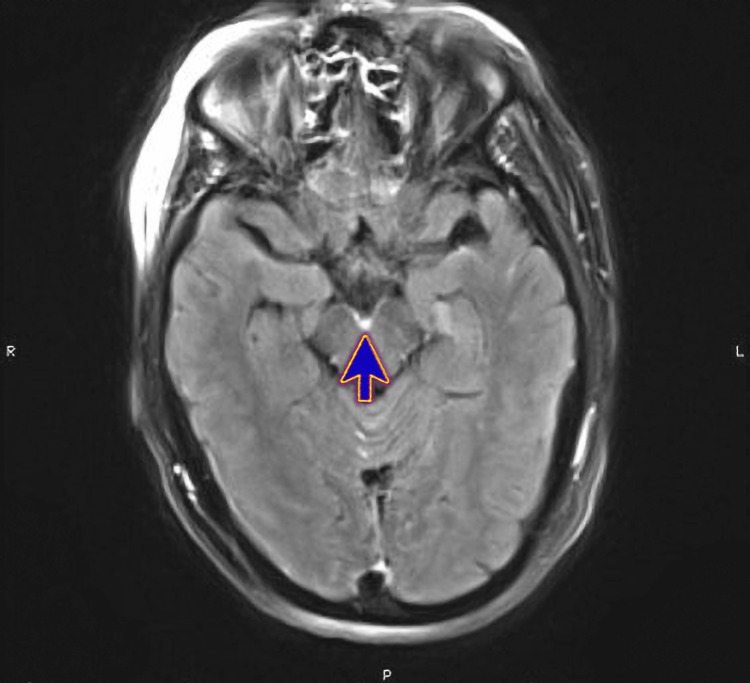
Magnetic Resonance Imaging (MRI) of the brain showing leptomeningeal enhancement along with interpedicular involvement with suspected cervical ependymal enhancement (blue arrow)

**Figure 3:**
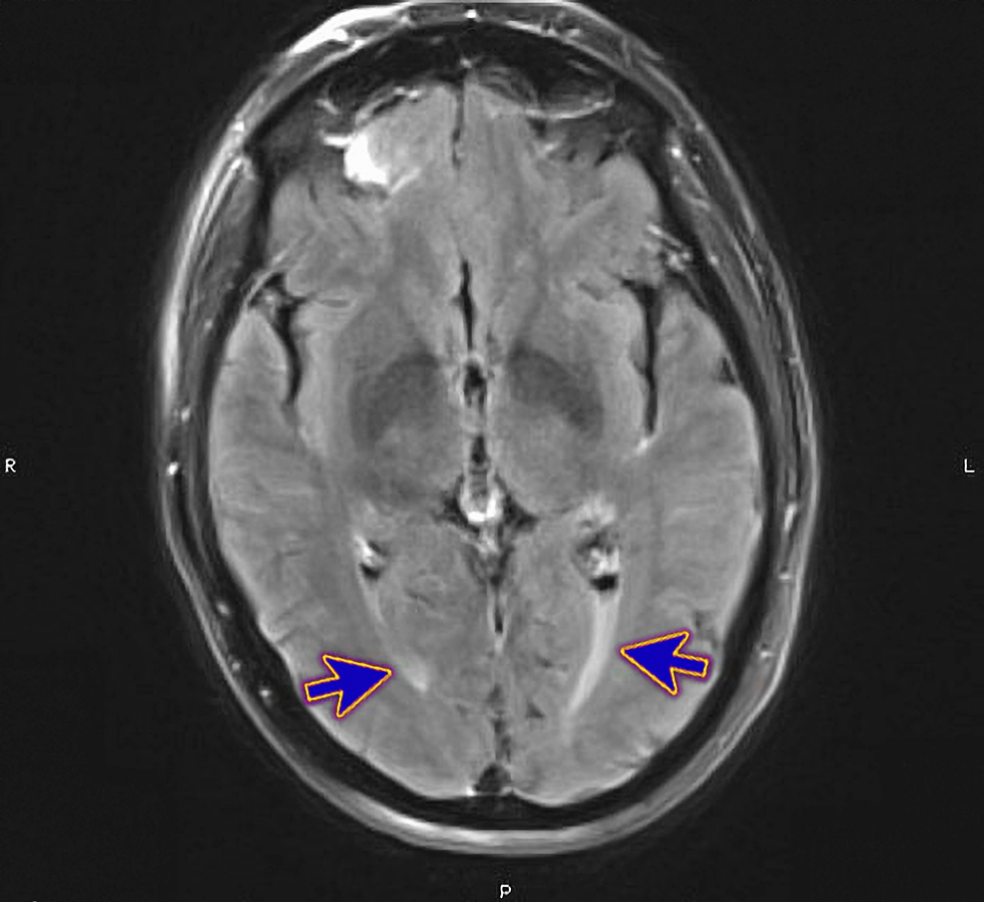
Magnetic Resonance Imaging (MRI) of the brain showing interval resolution of the sulcal and ventricular enhancement with resolved pus/ventriculitis (blue arrows)

Magnetic Resonance Imaging (MRI) of the brain revealed supratentorial and infratentorial leptomeningeal enhancement (Figure 1), infratentorial meningeal enhancement patch (Figure 2), ependymal enhancement, and pus in the lateral ventricles' dependent parts (Figure 3). These findings indicated meningoencephalitis, ventriculitis, and right sphenoidal sinusitis features.

Transthoracic echocardiography did not show signs of infective endocarditis. The patient continued receiving ceftriaxone 2 gm/day and showed substantial improvement by the third day, regaining normal consciousness. As the infectious disease specialist advised, she was discharged in good health and continued ceftriaxone for four weeks.

## Discussion

Meningoencephalitis is a grave condition precipitated by various infectious agents, encompassing bacteria, viruses, fungi, and parasites. A prominent member of the Mitis streptococci group, Streptococcus pneumoniae, is the most frequently encountered bacterial etiology of meningitis in adults [1]. Bacterial infections account for approximately 21.8% of all such cases, fungal and parasitic infections constitute 7.3%, while 17.2% are due to unidentified causes [2-4].

Another member of the same group, Streptococcus oralis, is predominantly in the oral cavity. It is generally characterized by its low pathogenic and virulent attributes, with a primary association with subacute infective endocarditis [5]. Nonetheless, there have been isolated instances, including nine documented cases before the present one, where this bacterium was identified as the inciting agent of meningoencephalitis, and our literature review disclosed several cases illuminating the varied clinical presentations associated with Streptococcus oralis-induced meningoencephalitis. Cardoso et al. reported a case concerning a 53-year-old man, where alcoholism and poor oral hygiene surfaced as the risk factors [6]. Similarly, Patel et al. described an instance of a 58-year-old female whose meningitis arose from a CSF leak due to right sphenoid meningoencephalocele and right temporal herniation [7]. Another fascinating case by Honda et al. emphasized a post-dental manipulation etiology in a 75-year-old man who developed symptoms merely four days after receiving dental treatment [8]. Lastly, Nakamura et al. illustrated the synergy of gingival bleeding and uncontrolled diabetes mellitus as the predisposing factors in an 81-year-old male [9].

In the present scenario, the patient manifested symptoms suggestive of meningitis, a diagnosis substantiated by the CSF analysis and MRI findings, which revealed meningoencephalitis and ventriculitis. Unlike many previously documented cases, our patient had no clear predisposing factor, such as dental procedures or other interventions. Furthermore, the rapid initiation of empirical treatment even before the culture results, based on clinical suspicion, might have played a crucial role in her rapid recovery. Additionally, while the CSF culture yielded no growth - a potential diagnostic challenge - our proactive approach led us to conduct a blood culture that identified the pathogen. This was crucial, given that a delay in diagnosis and treatment could have led to a more severe clinical outcome. Fortunately, the isolated pathogen was found to be susceptible to ceftriaxone, to which the patient demonstrated a favorable response, ultimately culminating in a complete recovery.

Given the known association of Streptococcus oralis with infective endocarditis, an echocardiogram was performed to exclude this diagnosis. The absence of any signs of endocarditis contrasted with several reported cases where concurrent endocarditis was identified.

This case underscores the imperative to include Streptococcus oralis in the differential diagnosis of meningitis or encephalitis, especially in immunocompromised individuals or those with pre-existing medical conditions [10]. Despite its customary role as a commensal organism in the oral cavity, it harbors the potential to induce severe infections under specific circumstances [11]. Moreover, this case accentuates the paramount importance of timely diagnosis and intervention in cases of meningitis or encephalitis. Expedited initiation of antibiotic therapy can significantly ameliorate outcomes and avert potential complications [12].

## Conclusions

This case underlines the importance of considering even common commensal organisms, such as Streptococcus oralis, as potential severe infection agents, especially in patients with pre-existing health conditions. It also highlights the necessity of immediate initiation of empirical antibiotic therapy, comprehensive diagnostic evaluation, and tailoring treatment based on culture and sensitivity results to optimize patient outcomes.

## References

[1] Sadowy E, Hryniewicz W (2020). Identification of Streptococcus pneumoniae and other Mitis streptococci: importance of molecular methods. Eur J Clin Microbiol Infect Dis.

[2] Dubot-Pérès A, Mayxay M, Phetsouvanh R (2019). Management of central nervous system infections, Vientiane, Laos, 2003-2011. Emerg Infect Dis.

[3] Mohan A, Munusamy C, Tan YC (2019). Invasive Salmonella infections among children in Bintulu, Sarawak, Malaysian Borneo: a 6-year retrospective review. BMC Infect Dis.

[4] El-Naggar W, Afifi J, McMillan D, Toye J, Ting J, Yoon EW, Shah PS (2019). Epidemiology of meningitis in Canadian neonatal intensive care units. Pediatr Infect Dis J.

[5] Wang A, Gaca JG, Chu VH (2018). Management considerations in infective endocarditis: a review. JAMA.

[6] Cruz Cardoso J, Ferreira D, Assis R, Monteiro J, Coelho I, Real A, Catorze N (2021). Streptococcus oralis meningitis. Eur J Case Rep Intern Med.

[7] Patel K, Memon Z, Prince A, Park C, Sajan A, Ilyas N (2019). Streptococcus oralis meningitis from right sphenoid meningoencephalocele and cerebrospinal fluid leak. BMC Infect Dis.

[8] Honda S, Inatomi Y, Yonehara T, Hashimoto Y, Hirano T, Uchino M (2006). [A case report of streptococcus oralis meningitis after dental manipulation]. Rinsho Shinkeigaku.

[9] Nakamura Y, Uemura T, Kawata Y, Hirose B, Yamauchi R, Shimohama S (2021). Streptococcus oralis meningitis with gingival bleeding in a patient: a case report and review of the literature. Intern Med.

[10] Sunnerhagen T, Nilson B, Olaison L, Rasmussen M (2016). Clinical and microbiological features of infective endocarditis caused by aerococci. Infection.

[11] Renton BJ, Clague JE, Cooke RP (2009). Streptococcus oralis endocarditis presenting as infective discitis in an edentulous patient. Int J Cardiol.

[12] Garcia HH, Nash TE, Del Brutto OH (2014). Clinical symptoms, diagnosis, and treatment of neurocysticercosis. Lancet Neurol.

